# How to Sleep

**Published:** 1854-02

**Authors:** 


### HOW TO SLEEP.

Sound, connected, early, refreshing sleep, is as essential to health as our daily food. There is no merit in simply getting up early. The full amount of sleep requisite for the wants of the system should be obtained, even if it requires till noon. I go to bed at nine o'clock the year round, and I stay there until I feel rested ; but I do not go to sleep again after I have once awaked of myself, after daylight. I remain in bed until the feeling of tiredness goes off, if there is any, and I get up when I feel like it. I do not sleep in the day-time ; it is a pernicious practice, and will diminish the soundness of repose at night. Dr. Holyoke, after he was a hundred years old, said, "I have always taken care to have a full proportion of sleep, which, I suppose, has contributed to my longevity." The want of sufficient sleep is a frequent cause of insanity. To obtain good sleep, the mind should be in a sober, quiet frame for several hours before bedtime. I think people require one hour's more sleep in winter than in summer. In connection with this subject, the North British Review illustrates the importance of sufficient sleep on a parallel with the natural history of the Sabbath :—"The Creator has given us a natural restorative—sleep ; and a moral restorative—Sabbath keeping; and it is ruin to dispense with either. Under the pressure of high excitement, individuals have passed weeks together with little sleep or none; but when the process is long continued, the over-driven powers rebel, and fever, delirium and death come on. Nor can the natural amount be systematically curtailed without corresponding mischief. The Sabbath does not arrive like sleep. The day of rest does not steal over us like the hour of slumber. It does not entrance us almost, whether we will or not; but, addressing us as intelligent beings, our Creator assures us that we need it, and bids us notice its return, and court its renovation. And if, going in the face of the Creator's kindness, we force ourselves to work all days alike, it is not long till we pay the forfeit. The mental worker—the man of business, or the man of letters—finds his ideas coming turbid and slow; the equipoise of his faculties is upset, he grows moody, fitful and capricious; and, with his mental elasticity broken, should any disaster occur, he subsides into habitual melancholy, or in self-destruction speeds his guilty exit from a gloomy world. And the manual worker—the artisan, the engineer—toiling on from day to day, and week to week, the bright intuition of his eyes gets blunted; and, forgetful of their cunning, his fingers no longer perform their feats of twinkling agility, nor by a plastic and tuneful touch, mold dead matter, or wield mechanic power; but mingling his life's blood in his daily drudgery, his locks are prematurely gray, his genial humor sours, and slaving it till he has become a morose or reckless man, for any extra effort, or any blink of balmy feelings, he must stand indebted to opium or alcohol."

A sleeping-room should be large and airy, the higher from the ground the better, even in the country; it should contain but very little furniture, no curtains or clothing of any description should be hung up in it, nor should it contain, for a moment, any vegetables or fruit, or flowers, or standing liquids of any kind; nor should there be any carpet on the floor, except a small strip at the side of the bed, so that in getting out of bed a shock may not be imparted by the warm feet coming in contact with the cold floor. The fire-place should be always left open during the day, for several hours; the windows and doors should be left open while the sun is shining, but the windows should be closed an hour or more before sundown. As soon as a person is dressed in the morning, he should leave his chamber; the bedding should be hung on chairs and allowed to air for several hours.

On going to bed, a window should be hoisted several inches at bottom, and, if practicable, be let down as much at top, that, while the heavy fresh air comes in below, the light and foul air may pass out above. As a general rule, it is far best to sleep in rooms where no fire has been burning since breakfast, but there should be bed-clothing enough to keep from feeling chilly. If it is bitter cold weather, with high winds, it may be better to build a moderate fire about dark, but not to let it go entirely out before morning. If there is any fire at all in a sleeping-room, it should not be allowed to go out altogether.

A person should sleep in one garment, a coarse cotton shirt, and no more, without a button, or pin, or string about him. No one, who pretends to common cleanliness, should sleep in a garment worn during the day, nor wear during the day a garment in which he has slept; any garment worn should have six or eight hours' airing every twenty-four hours.

No sleeping-room should be less than eight feet high, nor should it contain, for each person sleeping in it, less than one hundred and fifty feet superficial measure, or about twelve feet square.

To show what a bearing a small deficiency in the action of the lungs has on the health, I present the following calculation, applied to a night's sleep of eight hours:—A person in good health and of medium size will, in that eight hours' sleep, breathe nine hundred gallons of air; but if one-fifth of his lungs are inoperative, he consumes in the same time one hundred and eighty gallons less, and in the course of twenty-four hours, seven hundred gallons less than he ought to do. No wonder then that, when the lungs begin to work less freely than they ought to do, the face so soon begins to pale, the appetite fails, the strength declines, the flesh fades, and the victim dies. Not only are consumptions traceable to this habitual deficiency of respiration, but rheumatism, colds, chills, ague, bilious, yellow and putrid fevers, suppressions, whites, dyspepsia, and the like. So that, in every view of the case, any method which secures the prompt detection of this insufficient breathing, and rectifies it without delay, should merit and demand the immediate investigation of every lover of the health and happiness of mankind. *See next number.*

